# Heat stress affects fecal microbial and metabolic alterations of primiparous sows during late gestation

**DOI:** 10.1186/s40104-019-0391-0

**Published:** 2019-11-04

**Authors:** Jianwen He, Huiduo Guo, Weijiang Zheng, Yongqiang Xue, Ruqian Zhao, Wen Yao

**Affiliations:** 1 Laboratory of Gastrointestinal Microbiology, Jiangsu Key Laboratory of Gastrointestinal Nutrition and Animal Health, College of Animal Science and Technology, Nanjing Agricultural University, Nanjing, Jiangsu People’s Republic of China 210095; 2 National Experimental Teaching Center for Animal Science, College of Animal Science and Technology, Nanjing Agricultural University, Nanjing, Jiangsu People’s Republic of China 210095; 3 Key Laboratory of Animal Physiology and Biochemistry, Ministry of Agriculture and Rural Affairs of the People’s Republic of China, Nanjing Agricultural University, Nanjing, Jiangsu People’s Republic of China 210095

**Keywords:** Heat stress, Late gestation, Metabolic profiles, Microbial community, Sow

## Abstract

**Background:**

Heat stress (HS) jeopardizes intestinal barrier functions and augments intestinal permeability in pigs. However, whether HS-induced maternal microbial and metabolic changes in primiparous sows during late gestation remains elusive. We present here, a study investigating the fecal microbial and metabolic responses in late gestational primiparous sows when exposed to HS.

**Methods:**

Twelve first-parity Landrace × Large White F1 sows were randomly assigned into two environmental treatments including the thermoneutral (TN) (18–22 °C; *n* = 6) and HS (28–32 °C; *n* = 6) conditions. Both treatments were applied from 85 d of gestation to farrowing. The serum and feces samples were collected on d 107 of gestation, for analyses including intestinal integrity biomarkers, high-throughput sequencing metagenomics, short-chain fatty acid (SCFA) profiles and nontargeted metabolomics.

**Results:**

Our results show that HS group has higher serum Heat shock protein 70 (HSP70), lipopolysaccharide (LPS) and lipopolysaccharide-binding protein (LBP) levels. The gut microbial community can be altered upon HS by using β-diversity and taxon-based analysis. In particular, the relative abundance of genera and operational taxonomic units (OTUs) related to Clostridiales and *Halomonas* are higher in HS group, the relative abundance of genera and OTUs related to Bacteroidales and *Streptococcus*, however, are lower in HS group. Results of metabolic analysis reveal that HS lowers the concentrations of propionate, butyrate, total SCFA, succinate, fumarate, malate, lactate, aspartate, ethanolamine, β-alanine and niacin, whereas that of fructose and azelaic acid are higher in HS group. These metabolites mainly affect propanoate metabolism, alanine, aspartate and glutamate metabolism, phenylalanine metabolism, β-alanine metabolism, pantothenate and CoA biosynthesis, tricarboxylic acid cycle (TCA) and nicotinate and nicotinamide metabolism. Additionally, correlation analysis between significant microbes and metabolites indicated that the HS-induced microbiota shift is likely the cause of changes of intestinal metabolism.

**Conclusions:**

Taken together, we reveal characteristic structural and metabolic changes in maternal gut microbiota as a result of late gestational HS, which could potentially provide the basis for further study on offspring gut microbiota and immune programming.

## Background

Heat stress (HS) is a major environmental stressor that is detrimental to animal husbandry worldwide [1, 2]. Pigs are particularly sensitive to the hot environment due to lack of effective sweat glands [3]. Moreover, as the result of modern genetic selection, pigs have a thicker layer of subcutaneous adipose tissue that impedes radiant heat loss; the improved lean tissue accretion rate can increase endogenous heat production [3, 4]. The swine industry is severely affected by HS and the poor sow performance alone is estimated to lose over the US $450 million annually [5], and this economic loss could be further increased if the climate gets worse [6]. In our previous study, HS during late gestation augments sows’ rectal temperature, respiration rates, surface temperature and duration of eating. Moreover, it also exacerbates sows’ negative energy balance and enhances protein and lipid catabolism, which affects piglets’ daily creep feed consumption and their weaning weight [7]. These data indicate unique changes in sows’ health, nutrient digestion and energetic metabolism, which may be related to the altered gastrointestinal (GI) microbiota [8].

HS-mammals reallocate blood to the periphery in order to maximize radiant heat dissipation, and this blood redistribution is supported by the vasoconstriction of the GI tract [9]. As a consequence, the intestine receives lessened blood and nutrients that lead to hypoxia at intestinal epithelium, which eventually compromises its barrier and function [10]. The intestinal barrier is composed of the single layer of columnar epithelial cells joined together by the tight junction, serving as the body’s first line of defense against potentially harmful microbes and antigens residing within the intestinal lumen. This compromised intestinal integrity augments lipopolysaccharide (LPS) in the portal and systemic blood, contributing to multi-organ failure syndrome [11]. Meanwhile, this hypoxia at intestinal epithelium can impede nutrients digestion and absorption [12, 13]. Such compromised intestinal integrity and nutrients digestion may change the intestinal environment, which is home to trillions of microbes, namely gut microbiota. The gut microbiota is regarded as a forgotten organ in the host due to its capability to communicate with the host by fermenting host indigestible diet, which affects host physiological and immunological processes [14]. In particular, it has been suggested that the short-chain fatty acids (SCFAs), including acetate, propionate, and butyrate, act as crucial metabolites of gut microbiota and play diverse functions in shaping processes mentioned above [15]. Therefore, the alterations of gut microbiota are accompanied by the disruption of the symbiotic relationship between commensal microbiota and its host, and this symbiotic relationship is effortlessly demolished by stress that causing a variety of diseases [16–18].

In recent years, the effects of HS on microbial composition in broilers, laying hens, ducks, dairy goats and cows have been successively studied [19–23]. However, few studies have been reported on the effects of HS on the gut microbial ecosystem of pigs, particularly in the pregnant sow. Maternal gut microbiota can be transferred to the unborn fetus in utero through the placenta during pregnancy, affecting offspring immune programming [24, 25]. Hence, it is essential to better understand the composition and metabolic function of the gut microbiota under HS during pregnancy. These microbial and metabolic alterations during pregnancy may provide insights regarding how maternal HS influences offspring health via the gut microbiota. Herein, we present our study investigating the effects of HS during late gestation on gut microbiota and metabolism of sows, particularly in primiparous sows that are especially sensitive to HS [26]. Our study aims to reveal the adverse effects of HS on gut health of pregnant sows via microbial and metabolic analyses, and servers as a reference for a further study regarding maternal HS impact on offspring gut microbiota.

## Methods

### Animals, housing, experimental design, and sampling

Animals, housing and experimental design are detailed as described previously [7]. Briefly, twelve first-parity Landrace × Large White F1 sows were randomly assigned into two environmental treatments including the thermoneutral (TN) (18–22 °C; *n* = 6) and HS (28–32 °C; *n* = 6) conditions from 85 d of gestation until farrowing. The environmental treatments last about 30 d. Sows during late gestation were fed a corn-soybean meal-based diet twice daily and they were limit-fed by 3.0 kg/d without any feed left. Blood and fecal samples were collected at the week before farrowing (d 107 of gestation). Fasting blood samples were collected from sows using jugular venipuncture beginning at 09:00. A 10-mL blood collection tube with gel & clot activator (Kangjie equipment & supply Co., Ltd., Jiangsu, China) was used for serum collection. The blood sample was centrifuged at 3,000 r/min for 15 min at 4 °C, the serum was then collected and frozen at − 20 °C. Fecal samples were collected at 06:00 and immediately stored in sterile tubes and snap-frozen in liquid nitrogen before storage at − 80 °C for the further DNA extraction, SCFAs and metabolomic analysis. One TN sow was excluded from the further analysis because of only one stillbirth at farrowing.

### Serum biochemical parameters assays

Serum biomarkers of intestinal integrity and HS, including LPS, LPS-binding protein (LBP), intestinal fatty acid-binding protein (I-FABP), diamine oxidase (DAO) and heat shock protein 70 (HSP70) were determined by commercial enzyme-linked immunosorbent assay (ELISA) kit per the instructions (Fangcheng Beijing Technology Co. Ltd., Beijing, China). Assay sensitivities were above 0.01 EU/L, 1 ng/mL, 60 ng/L, 100 pg/mL and 10 pg/mL, respectively. The intra- and inter-assay coefficient of variations were 9% and 15%, respectively.

### DNA extraction, MiSeq sequencing, and bioinformatics analysis

The total genomic DNA was extracted from the fecal samples with a QIAamp Fast DNA Stool Mini Kit (QIAGEN, Hilden, Germany) and quantified with a NanoDrop spectrophotometer (Thermo Fisher Scientific Inc., Wilmington, DE, USA).

The V3-V4 regions of bacterial 16S rRNA gene were amplified using a universal forward primer 341F (5′-CCTAYGGGRBGCASCAG-3′) and a reverse primer 806R (5′-GGGACTACNNGGGTATCTAAT-3′). Purified amplicons were pooled in equimolar and paired-end sequenced (2 × 250) on an Illumina Miseq platform by Biozeron Biotechnology (Shanghai, China). The raw reads were deposited into the NCBI Sequence Read Archive database (Accession Number: SRP218775).

Raw sequence data generated from 16S rRNA Miseq sequencing were demultiplexed, quality-filtered using quantitative insights into microbial ecology (QIIME) (version 1.17). The 250 bp reads were truncated at any site receiving an average quality score < 20 over a 10-bp sliding window, discarding the truncated reads that were shorter than 50 bp. Exact barcode matching, two nucleotides mismatch in primer matching, reads containing ambiguous characters were removed, and only sequences that overlap longer than 10 bp were assembled in compliance with their overlap sequence. Reads which could not be assembled were discarded. Operational taxonomic units (OTUs) were clustered with 97% similarity cutoff using UPARSE (version 7.1, http://dirve5.com/uparse/), and chimeric sequences were identified and removed using UCHIME [27]. The phylogenetic affiliation of each 16S rRNA gene sequence was analyzed by RDP Classifier (http://rdp.cme.msu.edu/) against the SILVA (SSU119, https://www.arb-silva.de) 16S rRNA database using a confidence threshold of 70% [28].

We performed α- and β-diversity calculations and taxonomic community assessment here. Specifically, α-diversity including rarefaction analysis, the number of sequences, the number of observed OTUs, Ace and Chao richness estimators, and Shannon and Simpson diversity indices were assessed using MOTHUR [29]. Nonmetric multidimensional scaling (NMDS) plots based on the Bray-Curtis distance metric were used to visualize differences in β-diversity [30]. Additionally, the relative abundance at the phylum, genus, and OTUs levels were compared between the two groups, with levels higher than 1% within total bacteria defined as predominant, and sorted for comparison.

### Short-chain fatty acid concentrations analysis

The SCFA concentrations in the feces were determined by using a capillary column gas chromatograph (GC-14B, Shimadzu, Japan; Capillary Column: 30 m × 0.32 mm × 0.25 μm film thickness) described in a previous study [31].

### Sample preparation and GC-MS analysis

Fecal samples (100 mg) were transferred into 5-mL centrifuge tubes; 500 μL of dd H_2_O was added, and the tubes were vortexed for 60 s. An aliquot of 1000 μL methanol (containing 2-Chloro-*L*-phenylalanine (0.2 mg/mL) and Heptadecanoic acid (0.2 mg/mL) as internal quantitative standard) was added and vortexed for 30 s. The tubes were then placed into an ultrasound machine at 25 °C for 10 min, incubated on ice for 30 min, and centrifuged at 12,000 r/min at 4 °C for 10 min. The supernatant (1.2 mL) was transferred into a new 2 mL centrifuge tube. The samples were dried by vacuum concentration. Then, 60 μL of a 15 mg/mL solution of methoxyamine in pyridine was added into the dried extract, and the mixture was vortexed for 30 s and reacted for 120 min at 37 °C. The methoximation reaction was followed by adding 60 μL BSTFA reagent (containing 1% FMCS), which was reacted for 90 min at 37 °C and then centrifuged at 12,000 r/min and 4 °C for 10 min. Finally, the supernatant was transferred to a sample bottle for GC-MS analysis (Agilent 7890A/5975C, Agilent Technologies, Santa Clara, CA, USA).

The derivatized sample (1.0 μL) was immediately injected by an autosampler into an Agilent 7890A GC system coupled with an HP-5MS capillary column (5% phenyl:95% methylpolysiloxane, 30 m × 250 μm i.d., 0.25 μm film thickness; Agilent J & W Scientific, Folsom, CA, USA). Helium was used as the carrier gas at a constant flow of 1.0 mL/min through the column. The injection temperature was 280 °C, and the transfer line temperature and ion source temperature were set to 150 °C and 230 °C, respectively. The temperature ramp program was as follows: an initial temperature of 60 °C for 2 min, which was increased at 10 °C/min to 300 °C and held for 5 min. Mass spectrometry was performed using the full-scan method over the range from 35 to 750 *m*/*z*.

### GC-MS data processing and differential metabolites identification

After the raw data was collected, identification of the compounds was achieved by comparison of the mass spectrum and retention indices of all the detected compounds with their reference standards and database in the National Institute of Standards and Technology Library (http://srdata.nist.gov/gateway/) and Wiley Chemical Structure Library [32]. The relative quantitative peak areas of each detected peak were normalized to [^13^C_2_]-myristic acid, the stable isotope IS, and the data were arranged on a two-dimensional matrix consisting of arbitrary sample names (observations) and peak area (variables). The multivariate statistical analysis was conducted with the SIMCA-P+ version 13.0 software package (Umetrics, Umea, Sweden). The acquired GC/MS data were processed with orthogonal-partial least squares projection to latent structures and discriminant analysis (OPLS–DA). The metabolites with variable importance projection (VIP) values of 1.0 and *P*-values of 0.05 (threshold) were considered as metabolites that could discriminate between two dietary groups. The impact of HS on metabolic pathways and metabolite set enrichment analysis were evaluated based on an online tool (http://www.metaboanalyst.ca/faces/ModuleView.xhtml) [33].

### Statistical analysis

Power calculations identified a required sample size of six pigs per treatment group in order to enable detection of an effect size of 1.80 SD for microbial data with 80% power and a type I error of 5% by using G*Power Data Analysis [34]. Statistical analyses were performed using SPSS 25.0 software (IBM Inc. Chicago, IL, USA). α-diversity was analyzed by using the non-parametric Mann-Whitney U test. HSP70, Intestinal integrity biomarkers, SCFAs and metabolites were evaluated for normal distribution with the Shapiro-Wilk test. The Student’s t-test or the non-parametric Mann-Whitney U test was applied to compare the difference between the two groups. Data were expressed as mean ± SEM for the Student’s *t*-test or median for the non-parametric Mann-Whitney U test, and differences were considered statistically significant at *P* <  0.05. Analysis of similarity (ANOSIM) for multivariate data was performed using the “vegan” package in R (http://www.r-project.org/) for bacterial community structure comparison. Spearman’s rank correlation analysis between significantly changed bacteria and metabolite profiles was conducted using GraphPad Prism version 8.0 (GraphPad Software, San Diego, CA, USA). The correlation was considered significant at *P* <  0.05.

## Results

### Serum parameters analysis

We firstly explored the HSP70 level in the serum to determine whether the sows in our study were in HS induction successfully. Our result showed that HSP70 level in the serum was notably higher in the HS group (Table 1, *P* <  0.01), indicating that the pregnant sows in our study were indeed in HS status. Then, we investigated the HS effects on the intestinal integrity to determine whether the intestinal environment has been changed. Serum biomarkers of intestinal integrity, including LPS, LBP, I-FABP and DAO were measured. No differences were observed in serum IFABP and DAO levels as a result of late gestational HS, however, we found serum LPS and LBP levels were significantly higher (Table 1, *P* <  0.05).

**Table 1:** Effect of late gestational HS on the intestinal integrity biomarkers of primiparous sows

Items	Treatments		*P*-value
	TN	HS	
*n*	5	6	**–**
Serum HSP70, ng/mL	116.85 ± 9.65	152.17 ± 5.08	< 0.01
Serum LPS, EU/L	0.67 ± 0.04	0.83 ± 0.03	< 0.05
Serum LBP, ng/mL	22.64 ± 1.85	29.81 ± 1.06	< 0.01
Serum IFABP, ng/L	1350.02 ± 63.32	1354.32 ± 66.79	0.96
Serum DAO, pg/mL	5371.28 ± 489.01	5637.98 ± 246.08	0.62

Data are expressed as mean with standard error of mean (SEM)

### Fecal microbial community

**Fig. 1:**
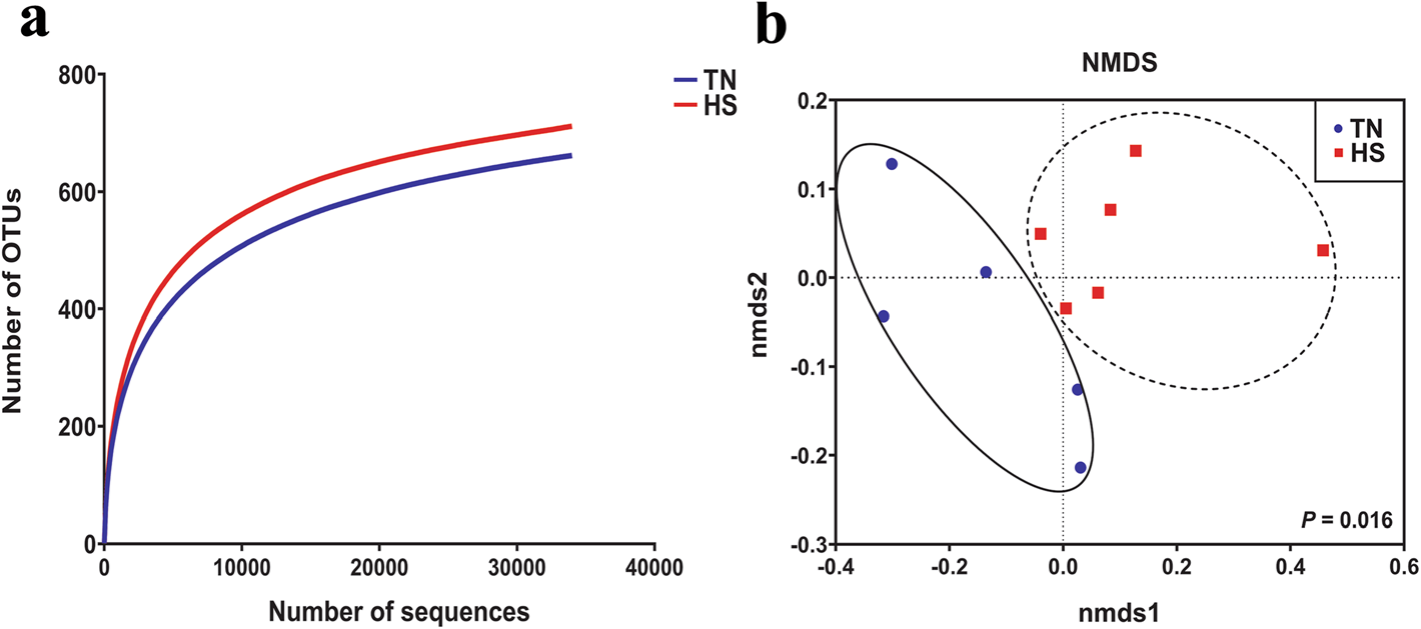
Effects of late gestational HS on the fecal microbiota of primiparous sows. **a** Rarefaction curves (mean curves for the samples/group) plotting the number of phylotypes found in the 16S rDNA gene libraries by the number of sequences from fecal microbiota of primiparous sows in the HS and TN groups. **b** Nonmetric multidimensional scaling (NMDS) ordination plots of fecal bacterial communities in the HS and TN group based on the Bray-Curtis distance metric. Circles with solid or dash line indicate that groups are significantly distinct using ANOSIM analysis (*P* < 0.05). HS = heat stress; TN = thermoneutral

To investigate HS effects on the composition of the gut microbiota, fresh fecal samples were collected individually from 6 HS-sows and 5 TN-sows during late stages (d 107) of gestation. The V3-V4 hypervariable region of bacterial 16S rRNA gene was amplified and sequenced for each sample using an Illumina Miseq PE250. Across all 11 samples, 496,343 high-quality sequences were classified as being bacteria with an average length of 416 bp. The rarefaction curves (mean curve for each group) showed that the majority of microbial diversity were sufficiently captured (Fig. 1a). The statistical estimates of α-diversity from each sample at a genetic distance of 3% are presented in Table 2. No effects were observed on any indices due to late gestational HS (Table 2), including the number of reads, OTUs, richness estimators (ACE and Chao1), and diversity indices (Shannon and Simpson). There were, however, a few noticeable changes in the community composition owing to HS. The Non-metric multidimensional scaling (NMDS) ordination plot based on the Bray-Curtis distance metric showed that the fecal bacterial communities in the samples were clearly separated by HS (Fig. 1b; ANOSIM, *P* = 0.016).

**Table 2:** Effect of late gestational HS on the richness and diversity of fecal microbiota in primiparous sows

Items	Treatments		*P*-value
	TN	HS	
*n*	5	6	**–**
Reads	43,410.00 ± 4374.00	43,626.00 ± 5035.00	0.98
OTUs	685.00 ± 51.00	730.70 ± 27.54	0.43
ACE^a^	756.20 ± 47.23	787.20 ± 25.29	0.56
Chao 1^a^	753.70 ± 46.72	797.20 ± 23.03	0.40
Shannon^b^	4.85 ± 0.16	4.95 ± 0.13	0.63
Simpson^b^	0.02 ± 0.00	0.02 ± 0.01	0.89

Data are expressed as mean with standard error of mean (SEM)

^a^Ace and Chao are the richness estimators that indicating the number of different species represented in an ecological community

^b^Shannon and Simpson are the diversity indices that estimating the diversity of an ecological community

**Fig. 2:**
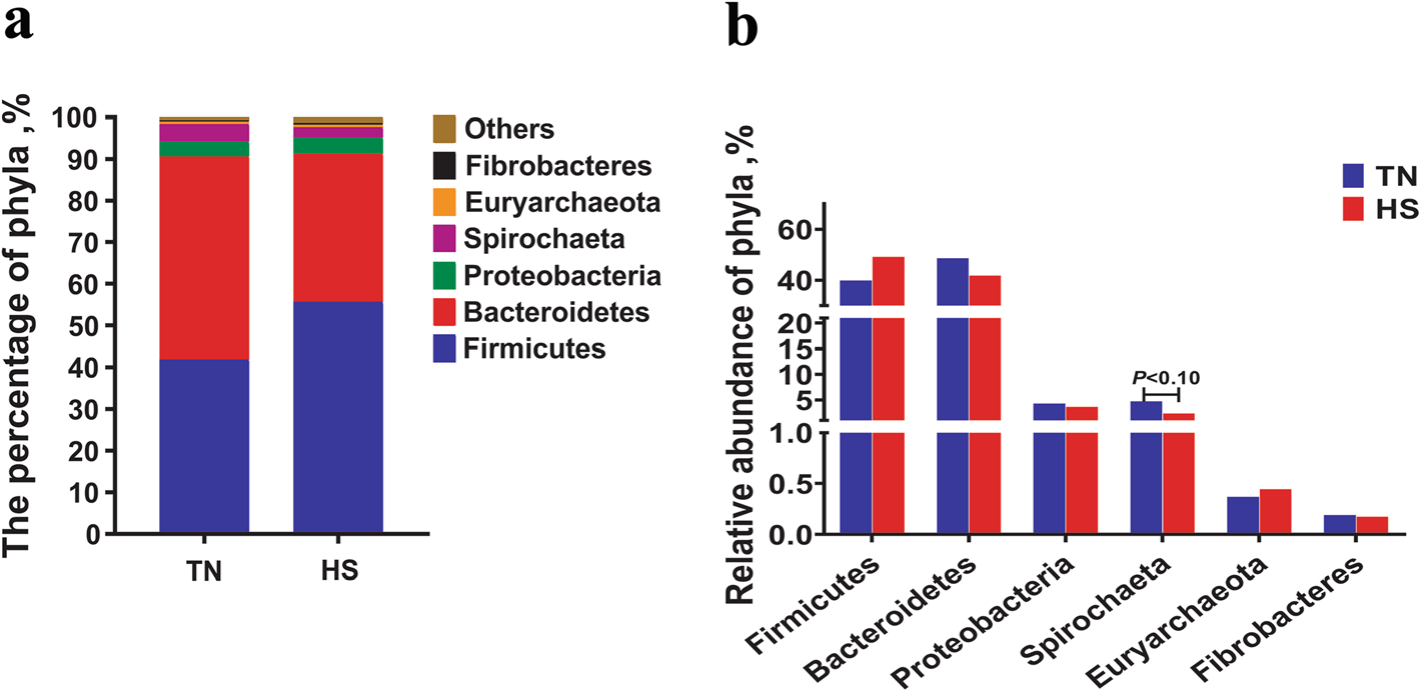
**a** Effects of late gestational HS on the fecal microbial composition at phylum in primiparous sows (relative abundance > 1%). **b** The statistical analysis of dominant phyla in the feces due to HS (relative abundance > 1%). The values were presented as the medians, with six or five sows per group. Statistical differences between two groups at each day were calculated by the Mann-Whitney U test. HS = heat stress; TN = thermoneutral

**Fig. 3:**
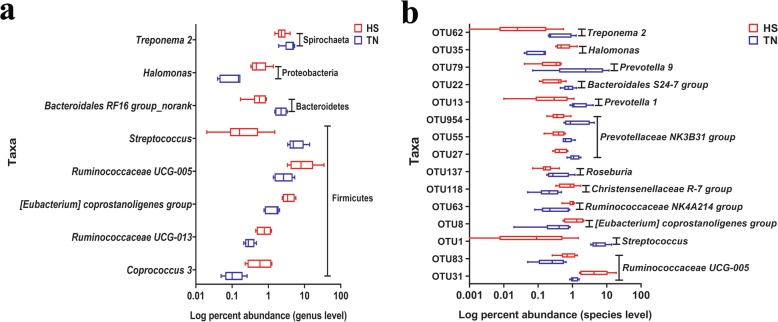
Effects of late gestational HS on the relative abundance of fecal dominant bacterial genera (**a**) and OTUs (**b**) in primiparous sows (relative abundance > 1%). The significantly changed genera and OTUs were presented (*P* < 0.05). The values were presented as the medians, with six or five sows per group. Statistical differences between two groups at each day were calculated by the Mann-Whitney U test. HS = heat stress; TN = thermoneutral

To further determine bacterial taxa that were responsible for the shift due to late gestational HS, bacterial taxa with a relative abundance of > 1% were subjected to taxonomic composition analysis. At the phylum level, Firmicutes and Bacteroidetes were the most predominant phyla in the feces, with a total relative abundance around 90%. The following rank is phyla Proteobacteria, Spirochaeta, Euryarchaeota, and Fibrobacteres (Fig. 2a). No statistical differences were observed in the relative abundance of the vast majority of phyla, such as Firmicutes, Bacteroidetes and Proteobacteria (Fig. 2b). However, HS tended to decrease the proportion of Spirochaetae (Fig. 2b, *P* = 0.08). The phylum-level analysis revealed that the gut microbiota composition of sows remained comparatively stable when exposed to late gestational HS. The genus-level analysis of the predominant abundant genera showed that *Ruminococcaceae UCG-005*, *[Eubacterium] coprostanoligenes group*, *Ruminococcaceae UCG-013*, *Coprococcus 3* and *Halomonas* were markedly higher in relative abundance by HS (Fig. 3a, *P* <  0.05), whereas the proportions of *Streptococcus* and *Bacteroidales RF16 group_norank* lowered sharply (Fig. 3a, *P* <  0.05), the decrease of *Treponema 2* was less significant (Fig. 3a, *P* <  0.10). Among these genera, *Streptococcus*, *Coprococcus 3* and *Halomonas* are significantly altered by an average of 17.54-fold, 5.57-fold and 5.31-fold, respectively. At the OTU level, 1004 OTUs were detected in the feces. As the results of HS, the relative abundance of OTUs closely related to *Streptococcus*, *Prevotella 1*, *Bacteroidales S24–7 group*, *Treponema 2* and *Prevotellaceae NK3B31 group* dramatically decreased (Fig. 3b, *P* <  0.05), and other OTUs related to *Prevotella 9*, *Prevotellaceae NK3B31 group* and *Roseburia* remained the same but are less remarkable (Fig. 3b, *P* <  0.10). In contrast, HS elevated the relative abundance of OTUs closely related to *[Eubacterium] coprostanoligenes group*, *Ruminococcaceae UCG-005*, *Halomonas*, *Ruminococcaceae NK4A214 group*, and *Christensenellaceae R-7 group* (Fig. 3b, *P* <  0.10).

### Fecal SCFA concentrations

To evaluate whether an alteration in the bacterial community structure would impact fermentability, concentrations of fecal SCFAs were determined. No effects of HS were found in the concentrations of acetate, isobutyrate, isovalerate, and valerate (Table 3, *P* > 0.05), whereas propionate, butyrate and total SCFA concentrations were observed to lower notably (Table 3, *P* <  0.05).

**Table 3:** Effect of late gestational HS on the fecal short-chain fatty acid (SCFA) concentrations of primiparous sows

Items	Treatments		*P*-value
	TN	HS	
*n*	5	6	**–**
Total SCFA	27.87 ± 3.12	20.02 ± 1.46	< 0.05
Acetate	11.11 ± 1.63	8.38 ± 0.65	0.18
Propionate	8.78 ± 0.99	5.74 ± 0.46	< 0.05
Butyrate	4.57 ± 0.44	3.01 ± 0.30	< 0.05
Isobutyrate	1.01 ± 0.06	1.02 ± 0.09	0.91
Valerate	0.50 ± 0.05	0.52 ± 0.03	0.71
Isovalerate	1.20 ± 0.09	1.36 ± 0.10	0.30

Data are expressed as mean with standard error of mean (SEM)

### Fecal metabolite profiles

**Fig. 4:**
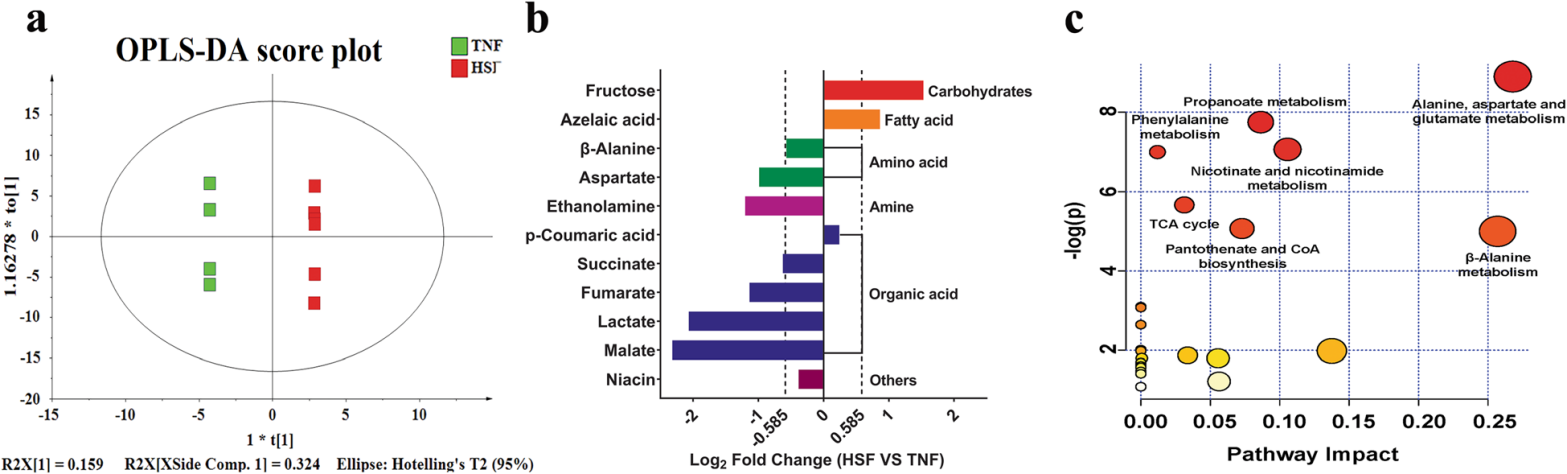
Effects of late gestational HS on the fecal metabolomic profiles of primiparous sows. **a** Orthogonal-partial least squares projection to latent structures and discriminant analysis (OPLS-DA) based on the fecal compounds data. The OPLS-DA score plots discriminating the feces of primiparous sows treated between TN (green) and HS (red) condition during late gestation [predictive ability parameter (Q^2^) (cum) = 0.813, goodness-of-fit parameter (R^2^) (X) = 0.872]. **b** Significant compounds. Metabolites accountable for class discrimination with *VIP* > 1, |log_2_ fold change*|* > 0.585, and *P* < 0.05 were listed. **c** metabolome view map of the differential metabolites (*VIP* > 1, *P* < 0.05) identified in the feces of primiparous sows treated between TN and HS condition during late gestation. The x-axis represents the pathway impact and the y-axis represents the pathway enrichment. The node color is based on its *P*-value, and the node radius is determined based on the pathway impact values. Larger sizes and darker colors represent higher pathway enrichment and impact values, respectively (For interpretation of the references to color in this figure legend, the reader is referred to the web version of this article). HS = heat stress; TN = thermoneutral

We detected a total of 172 nontargeted peaks and identified 85 metabolites in the fecal samples through GC/MS-based measurement. These metabolites can be divided into seven major groups, namely, amino acids, organic acids, phosphoric acid, carbohydrates, fatty acids, polyol, and amines, on the basis of the characteristics of each chemical. OPLS-DA model showed that there was an obvious separation in metabolites between the HS and TN group, suggesting that the fecal metabolic profiles have been considerably changed due to HS (Fig. 4a). More specifically, the relative concentrations of fructose and azelaic acid were elevated by HS (*VIP* > 1, log_2_ fold change> 0.585, and *P* <  0.05), whereas relative concentrations of malate, lactate, fumarate, succinate, ethanolamine, aspartate and β-alanine were lower in HS group (*VIP* > 1, log_2_ fold change<− 0.585, and *P* < 0.05) (Fig. 4b). Further metabolic pathways enrichment analysis indicated that late gestational HS mainly affected alanine, aspartate and glutamate metabolism, propanoate metabolism, phenylalanine metabolism, nicotinate and nicotinamide metabolism, TCA cycle, pantothenate and CoA biosynthesis, and β-alanine metabolism (Fig. 4c; *P* < 0.05).

### Correlations between fecal microbiota and metabolites

**Fig. 5:**
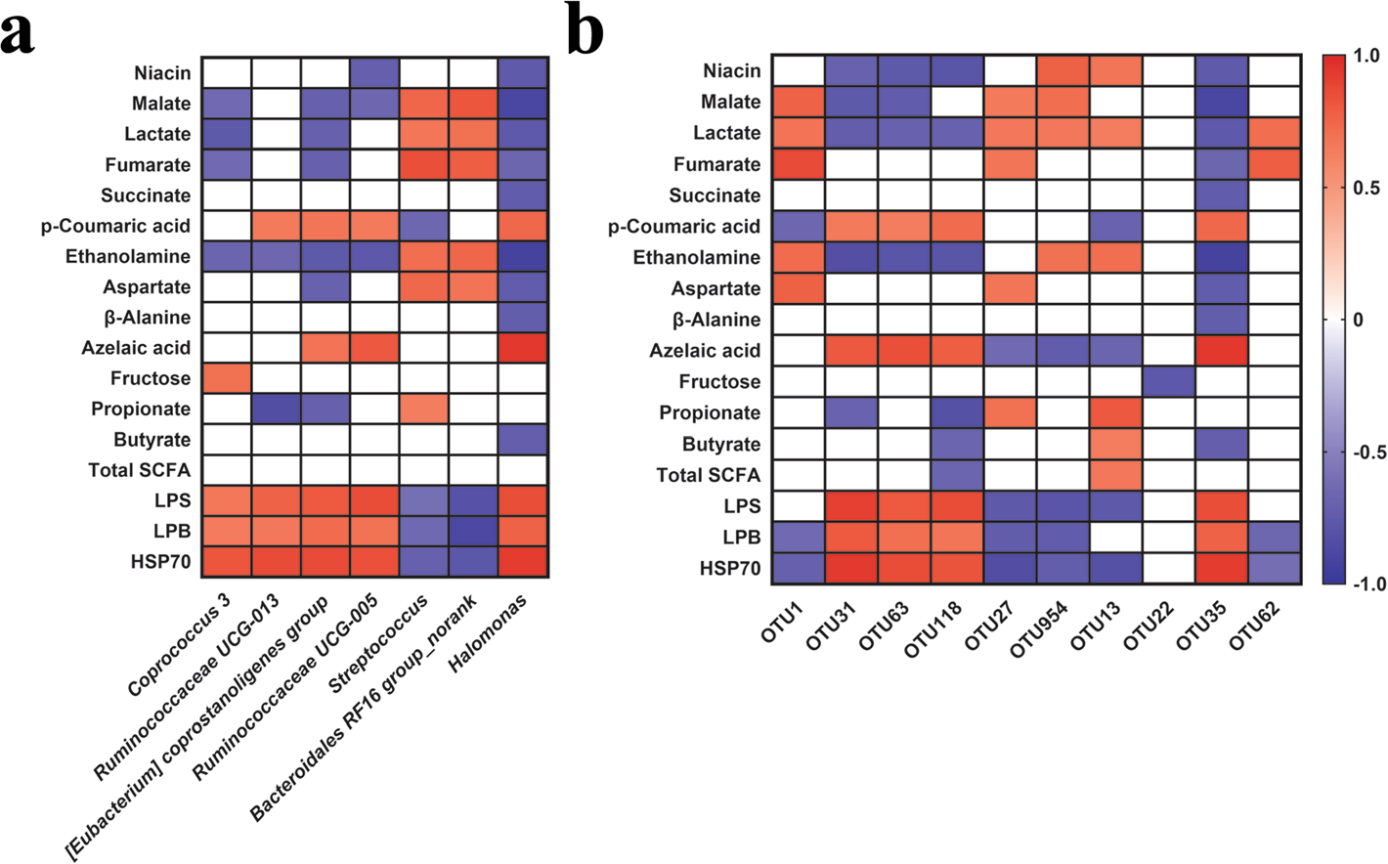
Effects of late gestational HS on correlation analysis between fecal bacteria (genus and OTU levels) and metabolite profiles (*P* < 0.05, *VIP* > 1). **a** Genus level. **b** OTUs level. The cells are colored based on the Spearman’s correlation coefficient between the significantly changed bacteria (relative abundance) and metabolites. Red represents significant positive correlation (*P* < 0.05), blue represents significantly negative correlation (*P* < 0.05), and white represents that the correlation was not significant (*P* > 0.05)

To investigate how the gut microbial community structure is related to different patterns of metabolites production due to altered physiologic status in HS, we analyzed the correlation between predominant taxa at both genus and OTU levels and significant fecal metabolites using the Spearman rank correlation coefficient (Fig. 5). At the genus level, the relative abundance of *Halomonas*, *[Eubacterium] coprostanoligenes group*, *Ruminococcaceae UCG-005*, *Coprococcus 3*, and *Ruminococcaceae UCG-013* were negatively correlated with the concentrations of organic acids (malate, lactate, and fumarate), amine (ethanolamine), amino acids (aspartate and β-alanine), SCFAs (propionate and butyrate), and niacin, but positively correlated with carbohydrate (fructose), fatty acid (azelaic acid), organic acid (p-coumaric acid), HSP70, LPS and LBP concentrations (Fig. 5a; *P* < 0.05). In contrast, the relative abundance of *Streptococcus*, and *Bacteroidales RF16 group_norank*, different from the former genera, showed the opposite correlations with significant metabolites (Fig. 5a; *P* < 0.05).

At the OTU level, the relative abundance of OTUs closely related to *Ruminococcaceae UCG-005* (OTU 31), *Ruminococcaceae NK4A214 group* (OTU 63), *Christensenellaceae R-7 group* (OTU 118) and *Halomonas* (OTU 35) were negatively correlated with the concentrations of organic acids (malate, lactate, and fumarate), amine (ethanolamine), amino acids (aspartate and β-alanine), SCFAs (propionate, butyrate and total SCFAs) and niacin, while positively correlated with organic acid (p-coumaric acid), fatty acid (azelaic acid), HSP70, LPS and LBP concentrations (Fig. 5b; *P* < 0.05). Conversely, the relative abundance of OTUs closely related to *Streptococcus* (OTU 1), *Prevotellaceae NK3B31 group* (OTU 27 and OTU 954), *Prevotella 1*(OTU 13), and *Treponema 2* (OTU 62) had the opposite correlations with significant metabolites (Fig. 5b; *P* < 0.05). Additionally, the relative abundance of OTUs closely related to *Bacteroidales S24–7 group* (OTU 22) was only inversely associated with carbohydrate (fructose) concentration.

## Discussion

To the best of our knowledge, the effects of HS on microbial composition in broilers, laying hens, ducks, dairy goats and cows have been reported successively in recent two years [19–23]. However, there is still unknown about the impact of HS on the pregnant sows’ microbial composition and its metabolites. A better understanding of the physiological alterations of microbial composition and its metabolites under HS could help to develop targeted approaches to prevent heat distress in pregnant sows. Therefore, we combined 16S rDNA sequencing and metabolomics technology to investigate the alterations of fecal microbial diversity and composition of pregnant sows, and the subsequent changes in fecal metabolites owing to late gestational HS. We report herein, HS during late gestation exerts a significant influence on both intestinal integrity and gut microbiota (microbial composition and metabolism).

As environmental heat-load increases, blood is diverted from the splanchnic organs to the skin by peripheral vasodilatation and GI tract vasoconstriction, orchestrating the distribution of blood away from the splanchnic bed [35]. Consequently, the intestinal epithelium can become hypoxic and hyperpermeable, and ultimately lead to endotoxemia, inflammation, and organ damage [9, 10]. In support of this, we observed higher serum LPS, LBP and HSP70 levels due to HS, demonstrating that HS causes compromised intestinal integrity and potential inflammatory state in pregnant sows [36]. The alterations of the physical gut barrier and immune function within GI microenvironments may be related to the shifts of the gut microbiota [37].

In the present study, late gestational HS affects the fecal microbial community structure of pregnant sows to a certain extent. Specifically, no significant differences were observed on α-diversity, including the OTUs, ACE, Chao, Shannon or Simpson index. Shannon and Simpson indices comprehensively reflect the species diversity and the community evenness, and the ACE and Chao indices demonstrate the bacterial species richness. These results are expected because it is not easy for HS to influence microbial richness and evenness, which has been reported to remain stable during pregnancy [38–40]. However, analysis of β-diversity indicates that the fecal bacterial communities in the samples are clearly separated by HS based on the Bray-Curtis distance metric. To the best of our knowledge, our results firstly demonstrate HS alters the microbial community structure in pregnant sows.

Including the microbial structure changes, we further analyze the microbial composition in HS and TN sows. Although the composition of the core gut microbiota is thought to be essentially stable throughout adulthood, some components are dynamic, biologically and metabolically flexible to environmental stresses by alteration in species composition that may influence health or disease risk [41]. Similar to the previous studies, our results also indicate that Firmicutes and Bacteroidetes are the two most predominant phyla, followed by Proteobacteria, Spirochaetes, and Euryarchaeota in pregnant sows [30, 42]. Except for the lower Spirochaetes, no other dominant phyla are altered by HS. The results are inconsistent with those of the previous report in heat-stressed growing pigs [43], the reasons for the differences are unconfirmed but we speculate that microbial composition is more stable at phylum level in pregnant sows [38–40]. Although no statistical differences are observed, HS increases the relative abundance of Firmicutes by 14% while decreasing Bacteroides by 13%. The increased ratio of Firmicutes to Bacteroidetes may facilitate extracting energy from food and stimulate lipogenesis in the case of energy supply [44]. This result is also supported by the exacerbated negative energy balance in heat-stressed pregnant sows reported in our previous study [7]. The gut microbiota is inclined to alter its composition to ingest energy more completely from food when the host is in the state of negative energy balance. Moreover, there are some interesting findings based on the analyses of genus and OTUs levels. Our results reveal that HS principally changes some genera related to Clostridiales and Bacteroidales. Particularly, HS elevates the relative abundance of most Clostridiales genera except for *Roseburia*, including Ruminococcaceae, *[Eubacterium] coprostanoligenes group*, *Coprococcus*, and Christensenellaceae. In contrast, it lowers the proportions of several Bacteroidales genera, including *Bacteroides* and *Prevotella*. It is well known that both Clostridiales and Bacteroidales act as the crucial SCFAs producers, which are beneficial to the gut development and the maintenance of gut homeostasis [15, 45, 46]. Taken together, the SCFAs data unveils that HS decreases the concentrations of propionate, butyrate and Total SCFAs, we speculate that the lower Bacteroidales genera and *Roseburia* may play a major role in the decline of SCFAs owing to HS in our study. Besides, HS promotes the some Clostridiales genera levels, which may be related to its capacity on protecting host from pathogen infection and abrogating intestinal pathology upon pathogen challenge [47]. We speculate that the higher Clostridiales should be self-protection of the GI tract under HS. Additionally, HS also lowers the relative abundance of *Streptococcus* while enhances the relative abundance of *Halomonas*. *Streptococcus* has two main roles. Firstly, it has the capacity to synthesize acetate via the Wood-Ljungdahl pathway, which will further synthesize butyrate [48, 49]. The lowered *Streptococcus* may also contribute to the decreased concentration of butyrate. Secondly, *Streptococcus* is a kind of urea-decomposing bacteria, which can degrade nitrogen compounds into ammonia via producing urease [50]. The lowered *Streptococcus* may be associated with a decreased amount of ammonia (ethanolamine) in the hindgut. The greater capacity of absorption for nitrogen compounds are related to our previous study that HS induces protein catabolism in pregnant sows [7]. *Halomonas* is reported as an opportunistic pathogen and may display pathogenic potential [51]. Its augment may suggest an enlarged risk of infection in the gut of HS pregnant sows.

Given the significant differences in fecal microbiota, we next investigated the alterations of fecal metabolites. Fecal metabolites indicate the final status of animal digestion, absorption, and metabolism [52]. Dietary fibers, protein and peptides, which escape digestion by host enzymes in the upper gut, are metabolized by the microbiota in the hindgut [53]. In our study, two major SCFAs, propionate and butyrate, are lower due to late gestational HS. Propionate can be formed from phosphoenolpyruvate (PEP) through the succinate pathway or the acrylate pathway, in which succinate or lactate acts as precursors [54]. Our metabolome data show that HS decreases the concentrations of lactate, malate, fumarate and succinate, which are intermediate products of bacterial synthesis of propionate. Moreover, fructose level is observed to be higher, suggesting that HS lowers monosaccharide absorption in pregnant sows, and further adversely affects both acrylate and succinate pathways via pyruvate for propionate biosynthesis. The metabolic pathway enrichment analysis also shows that propanoate metabolism pathway is affected. It is well known that propionate is carried by the bloodstream to a variety of different organs, where they are used as substrates for oxidation, lipid synthesis, and energy metabolism. Particularly, the hepatocyte cells of the liver can use propionate for gluconeogenesis [55, 56]. The decreased propionate level in our study may affect the functions discussed above. Butyrate is synthesized from the condensation of two molecules of acetyl-CoA and subsequently reduced to butyryl-CoA, which can be transformed to butyrate by butyryl-phosphate or via the butyryl-CoA: acetate CoA-transferase route [49, 57]. Some microbes also synthesize butyrate via lactate and acetate [15]. Our metabolome data indicate that HS decreases the concentrations of lactate and β-alanine. β-alanine can be diverted into pantothenic acid and coenzyme A biosynthesis, and further impact the level of acetyl-CoA. This result is also supported by the metabolic pathway enrichment analysis that β-alanine metabolism and pantothenate and CoA biosynthesis pathways are affected due to HS. The diminished butyrate may affect host physiological status negatively, including disturb neutrophil function and migration, decrease tight junction proteins expression in colon epithelial, and reduce anti-inflammatory effects by diminishing cytokine and chemokine release from immune cells [8]. Additionally, the concentrations of aspartate and ethanolamine were observed to be decreased owing to HS in our study. Aspartate is the precursor to several essential amino acids (EAAs) for animals, including methionine, threonine, isoleucine, and lysine [58]. The lowered aspartate implies an increase of its conversion to these essential amino acids. Since no significant differences are observed in these amino acids, we speculate that the capacity of dietary protein digestion, absorption, and metabolism may be enhanced in the HS group. This conjecture is consistent with our previous study that late gestational HS exacerbates the negative energy balance of sows, and the enhanced dietary protein availability may partially compensate for this scarcity. Meanwhile, the elevated digestion and absorption of dietary protein in the small intestine can decrease the fermentation of amino acids by large intestinal microbiota, leading to a lessened level of ethanolamine in the hindgut [59]. In favor of this, our metabolic pathways enrichment analysis revealed that alanine, aspartate and glutamate metabolism and phenylalanine metabolism pathways are changed.

The gut microbiota act in a coordinated manner to achieve metabolic communication with the host [8], we explored the correlations between the differential genera and OTUs and 17 major metabolites. It is noteworthy that most genera and OTUs related to are negatively correlated with propionate and butyrate or their intermediated metabolites (malate, lactate, fumarate). Such contradictory results are perhaps comprehensible since the greater relative abundance of these bacteria may alleviate the adverse effects of HS on the GI tract and promote the SCFAs production for improving gut homeostasis. Moreover, genera and OTUs related to Bacteroidales are positively correlated with propionate and butyrate or their intermediated metabolites (malate, lactate, fumarate), while being negatively associated with the LPS and LBP. LPS are immunogenic molecules derived from Gram-negative bacteria that can enter the bloodstream, causing an inflammatory response, and they are usually used for the evaluation of gut barrier function. Also, LPS and HSP70 are significant biomarkers of HS, and their high concentrations are usually observed in animals due to HS [19, 60, 61]. These results suggest that the lower Bacteroidales may be related to the disrupted gut homeostasis and enhanced inflammatory response. *Halomonas* is a crucial genus in our study that takes correlations with most differential metabolites due to HS. Specifically, it is negatively correlated with the SCFAs biosynthesis and nitrogen degradation (ethanolamine), while positively associated with the HSP70 and LPS levels. Given its positive response to HS and complex role in metabolism, we hypothesize that *Halomonas* may have the potential to act as a biomarker in the GI tract during gestational HS. This potential will be verified in further study. *Streptococcus*, as Gram-positive bacteria, is also positively associated with SCFAs biosynthesis and nitrogen degradation as we discussed above.

## Conclusions

In conclusion, based on the changes in the circulating levels of HSP70 and LPS, HS-induced pregnant animal model is successfully established. Our study here indicates that late gestational HS causes profound changes in the gut microbial composition, especially in the abundance and diversity of some SCFAs-producing species. These bacteria subsequently alter the SCFA formation and nitrogen degradation and further influence the gut homeostasis and inflammatory response. Our results suggest that HS-induced sows’ performance may be mediated in part by altered gut microbiota and metabolism, which may further affect offspring gut microbiota and immune programming. Additionally, it is noteworthy that *Halomonas* presents a vital role in the metabolism owing to HS, and its potential as an HS indicator should be validated in further study.

## Data Availability

The datasets used and/or analyzed during the current study are available from the corresponding author on reasonable request.

## Abbreviations

ANOSIM: Analysis of similarity for multivariate
CoA: Coenzyme A
DAO: Diamine oxidase
ELISA: Enzyme-linked immunosorbent assay
GC-MS: Gas chromatography-mass spectrometry
GI: Gastrointestinal
HS: Heat stress
HSP70: Heat shock protein 70
I-FABP: Intestinal fatty acid-binding protein
LBP: LPS-binding protein
LPS: Lipopolysaccharide
NMDS: Nonmetric multidimensional scaling
OPLS-DA: Orthogonal-partial least squares projection to latent structures and discriminant analysis
OTUs: Operational taxonomic units
QIIME: Quantitative insights into microbial ecology
SCFA: Short-chain fatty acid
TCA cycle: Tricarboxylic acid cycle
TN: Thermoneutral
VIP: Variable importance projection
